# Prediction Model with High-Performance Constitutive Androstane Receptor (CAR) Using DeepSnap-Deep Learning Approach from the Tox21 10K Compound Library

**DOI:** 10.3390/ijms20194855

**Published:** 2019-09-30

**Authors:** Yasunari Matsuzaka, Yoshihiro Uesawa

**Affiliations:** Department of Medical Molecular Informatics, Meiji Pharmaceutical University, Tokyo 204-8588, Japan; matsuzys@my-pharm.ac.jp

**Keywords:** chemical structure, constitutive androgen receptor (CAR), DeepSnap, Tox21, deep learning, QSAR

## Abstract

The constitutive androstane receptor (CAR) plays pivotal roles in drug-induced liver injury through the transcriptional regulation of drug-metabolizing enzymes and transporters. Thus, identifying regulatory factors for CAR activation is important for understanding its mechanisms. Numerous studies conducted previously on CAR activation and its toxicity focused on in vivo or in vitro analyses, which are expensive, time consuming, and require many animals. We developed a computational model that predicts agonists for the CAR using the Toxicology in the 21st Century 10k library. Additionally, we evaluate the prediction performance of novel deep learning (DL)-based quantitative structure-activity relationship analysis called the DeepSnap-DL approach, which is a procedure of generating an omnidirectional snapshot portraying three-dimensional (3D) structures of chemical compounds. The CAR prediction model, which applies a 3D structure generator tool, called CORINA-generated and -optimized chemical structures, in the DeepSnap-DL demonstrated better performance than the existing methods using molecular descriptors. These results indicate that high performance in the prediction model using the DeepSnap-DL approach may be important to prepare suitable 3D chemical structures as input data and to enable the identification of modulators of the CAR.

## 1. Introduction

The Toxicology in the 21st Century (Tox21) project is a multi-agency collaboration consortium, constituted of the National Institutes of Health, the US Environmental Protection Agency, the National Toxicology Program, the National Center for Advancing Translational Sciences, and the Food and Drug Administration, which focuses on developing and evaluating novel efficient methods for toxicity assessments and mechanistic insights, in addition to reducing time, costs, and the use of animals [1,2]. To establish the effects of agonist and antagonist models on toxicity pathways by utilizing a quantitative high-throughput screening based on in vitro human cell-based assays [2,3,4], it has profiled a library in the program including more than 10,000 chemical compounds, such as commercial chemicals, pesticides, food additives, and drugs, referred to as the Tox21 10K library. In the Tox21 program, the fundamental concept is based on an adverse outcome pathway (AOP), which is a structured representative idea of biological events in a linear way. This links a series of causally connected key events (KEs) between a molecular initiating event (MIE), triggered by environmental pollutants, drugs, food additives, and pesticides, and an adverse outcome (AO), including organ toxicity, such as hepatotoxicity, and toxicological endpoints, such as mutagenicity, carcinogenicity, cytotoxicity, immunotoxicity, and neurotoxicity, whose linkage is specially called by key event relationships (KERs) [5,6,7,8,9,10,11,12,13,14,15,16]. By considering the MIEs for developing toxicity, a broad range of important genes in their signaling pathways, such as a nuclear receptor, stress response, genotoxicity, and cell death, have been selected in the Tox21 project [17,18,19,20]. Among them, the *constitutive androstane receptor* (*CAR nuclear receptor subfamily 1*, *group I*, *member 3*, *NR1I3*) is a member of the nuclear receptor superfamily usually expressed in the liver. Additionally, the CAR is a ligand-activated transcription factor, which regulates the transcriptions of cellular responses and involves not only the metabolism and transport of numerous xenobiotics and endogenous chemicals, but also energy metabolism, tumor propagation, and cancer therapy for coordinated detoxification systems [4,21,22,23]. Despite the biological importance, the construction of in silico models to predict *CAR* activity with chemical compounds is possibly disturbed by the constitutive activity based on the normal condition, in which it is difficult to identify the important structural characteristics for ligand binding [24,25]. Furthermore, for the data, most toxicological assessments for these novel chemical compounds with agonist or antagonist activities rely on animal models, which are expensive, time-consuming, and difficult to translate into a human being. Thus, a potential alternative approach is required to the traditional animal toxicity evaluations [26,27]. Although the in silico approaches have long been the focus of interest as an alternative to animal testing, the conventional machine learning (ML) approaches, such as random forest (RF), support vector machine (SVM), and neural networks (NNs), for building precise prediction models require feature selection to avoid the combinatorial optimization problem and find a suitable combination, and feature learning to reduce overfitting by too many molecular descriptors with complicated craftsmanship skills, which is time-consuming [28,29,30]. Furthermore, a prediction model using two-dimensional (2D) descriptors may have difficulty recognizing the differences resulting from molecular chirality [31]. Thus, by utilizing molecular descriptors, the automated classification of chemical compounds may not be so easy due to the difficulty of feature extraction.

Recently, it was proven that deep learning (DL), which is an ML algorithm based on a convolutional neural network (CNN), can successfully and automatically capture the spatial and temporal dependencies in an image through the application of relevant filters, the latter are useful tools to build high-performance prediction models due to their powerful feature discrimination capability [32,33,34,35]. Numerous studies have been conducted on the prediction performance between the DL and the conventional ML methods, and these have shown better performance in the DL than in the conventional ML methods, indicating that the DL method may enable the building of a prediction model, such as chemical toxicity [36,37,38,39]. One important perspective on the high performance of the prediction models is access to large datasets, in which the DL method uses transfer-learning techniques, defined as a system to recognize and apply the knowledge learned in a previous task. This was shown to improve the performance in proportion to the number of input datasets by applying regularization techniques, such as dropout, early stopping, and weight decay [40,41]. In addition, another is the quality of the data sources. If the imprecise representation of chemical structures and inaccurate information of activity were utilized to design a prediction model, a large chemical space may indicate activity cliffs and a discontinuous region in a structure or activity surface [38,40,42,43]. Thus, the performance of the DL method may depend on the quantity and the quality of the input datasets. Moreover, the DL approach has not established the preparation of suitable input data of chemical structures. Thus, a novel capturing technique of their molecular features from molecular structure input data has been reported [44]. In this study, three-dimensional (3D) optimized molecular structures, which can be rotated at any arbitrary angle on the x-, y-, and z-axes, were photographed as a ball-and-stick model with different colors to represent the corresponding atoms to automatically input as much structural information as possible into the DL models. This is called DeepSnap [44]. When compared with the performance of the state-of–the–art methods using RF and DL with 3D or extend-connectivity fingerprint descriptors, the performance of the DL prediction model using the image input data produced by DeepSnap indicated that the receiver operating characteristic (ROC)-area under the curve (AUC) value in the DeepSnap-DL approach outperformed [44]. In addition, in the DeepSnap technique, the optimization of some parameters has been shown to involve optimal thresholds to obtain the best performance of the prediction models [45]. Furthermore, the studies conducted previously on the DeepSnap-DL method were insufficient for the prediction performance. Given the remarkably high performance of CNNs with promising robustness and self-learning capability [46,47,48], it could be hypothesized that the DeepSnap-DL technique using a DL algorithm causes the prediction models to have a high performance and high throughput for chemical compounds on toxicity.

Therefore, this study aimed to assess input datasets and parameters in the DeepSnap-DL method and investigated how they affect the prediction performance for CAR activity. Herein, the combinations of the various cleaning rules, such as adjusting protonation states and coordinating washed species in washing a molecular operating environment (MOE) database, were investigated. Consequently, when chemical structures produced by CORINA were applied as input data in the DeepSnap-DL method, very high performance of the prediction model was obtained and the calculation cost was reduced. These results indicate that conformational sampling is important in building of the high-performance prediction model in the DeepSnap-DL approach.

## 2. Results and Discussion

### 2.1. Contributions of Parameters for Prediction Performance in the DeepSnap-DL Approach

**Figure 1:**
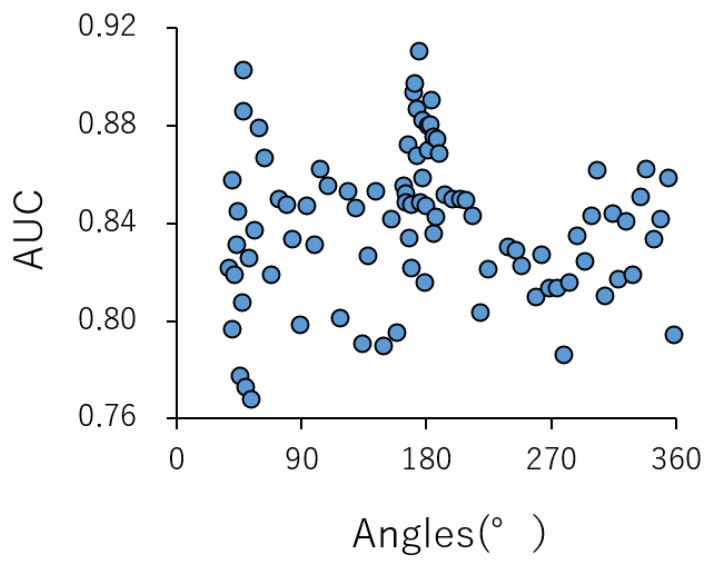
A contribution of performance of prediction models with angles of production of pictures in the DeepSnap approach: area under the curve (AUC), which was calculated by the deep learning (DL) build prediction models in GoogLeNet using training, validation, and external test datasets produced by the DeepSnap approach with 92 and 53 different angles from (360°, 360°, 360°) to (38°, 38°, 38°) and from (360°, 360°, 360°) to (90°, 90°, 90°), with MPS: 100, ZF: 100, AT: 23%, BR: 21.1 mÅ, BMD: 0.4 Å, BT: 0.8 Å, LR: 0.01, and BS: default.

We have previously investigated that the optimal ranges of parameter values in the DeepSnap-DL process attained the high performance of the prediction models for CAR activity [45]. In addition, in order to analyze the influence of different splits for Tra, Val, and Test datasets, we randomly divided the input data of a total of 7,141 chemical compounds into 32 kinds of ratios, that is, Tra:Val:Test = 1:1:1 to 28:28:1(Table S1, Figure S1), and then built a total of 32 prediction models by applying the DeepSnap-DL approach (Table S2). The results, for average loss (Tra), loss (Val), Acc (Val), AUC, and BAC were 0.192 ± 0.160, 0.216 ± 0.013, 91.80 ± 0.56, 0.785 ± 0.030, and 0.803 ± 0.057, respectively (Table S2). Moreover, the minimum loss (Tra), loss (Val), maximum Acc (Val), AUC (Test), and BAC (Test) were 20:20:1, 12:1:1, 8:1:1, 26:26:1, and 26:26:1, respectively, in the ratio of Tra:Val:Test (Table S2). Therefore, we combined the ratio of Tra:Val:Test (26:26:1) for the maximum AUC, while the BAC was selected for the next analysis. To investigate the contributions of the angles in capturing Jmol-generated images in the DeepSnap approach to predict the performance in detail, the angles on the *x*-, *y*-, and *z*-axes were optimized using a total of 7141 nonoverlapped chemical compounds consisting of 768 active and 6373 inactive compounds, which were prepared by eliminating the same chemical structures with different annotation numbers (Table S3). The 3D chemical structures were curated and optimized to generate a single low-energy conformation utilizing the CORINA classic software in the MOE modeling program. A total of 92 different angles on the *x*-, *y*-, and *z*-axes from (360°, 360°, 360°) to (38°, 38°, 38°) (Table S4), which produced from one to one thousand pictures from the 3D structures in the DeepSnap approach, were assessed for the prediction performance using the following datasets ratio: Tra:Val:Test (26:26:1). In this study, the mean values of the AUC and the BAC for the 92 angles used were 0.839 ± 0.031 and 0.789 ± 0.030, respectively (Figure 1, Figure S2). The maximum values of the AUC and the BAC for the angles were 0.910 and 0.867 at angle 176°, respectively (Figure 1, Figure S2). These results indicated that there is an appropriate angle that demonstrates high prediction performance. Furthermore, to assess the contribution made by the depiction of the chemical structures to the prediction performance, the BR of parameters in the DeepSnap process were examined.

By employing the nonoverlapped chemical compounds in the DeepSnap approach, we studied a total of 101 kinds of BR chemical compounds in Jmol-generated ball-and-stick structure models, including from 3 to 30 mÅ at angle 176°. The mean AUC and BAC values for the 101 kinds of BRs applied in this study were 0.871 ± 0.021 and 0.813 ± 0.043, respectively (Figure S3a,b). The maximum AUC and BAC values for the BRs demonstrated 0.914 at a BR:14.5 mÅ and 0.879 at a BR:17.2 mÅ, respectively (Figure S3a,b). Moreover, to investigate the contributions of hyperparameters in the DL to the prediction performance, a total of 68 batch sizes (BSs) from 1 to 600, and 250 learning rates (LRs) from 0.036 to 0.00002 were fine-tuned using the nonoverlapped chemical compounds. In this study, the mean AUC and the BAC values for 68 kinds of BSs or 250 kinds of LRs used were 0.882 ± 0.019 (AUC for BS) and 0.816 ± 0.054 (BAC for BS) (Figure S3a,b) or 0.885 ± 0.021 (AUC for LR) and 0.829 ± 0.027 (BAC for LR) (Figure S3c,d), respectively. The maximum AUC and BAC values for the BS or LR were 0.918 (AUC at BS:108) and 0.867 (BAC at BS:11) (Figure S4a,b) or 0.925 (AUC at LR:0.00061) and 0.881 (BAC at LR:0.00062), respectively (Figure S3c,d). Besides, to assess the contributions made by the two DNNs, AlexNet and GoogLeNet, with parameters in DL, the AUC and BAC were calculated for the total 92 kinds of BSs in these two DNNs. For the AlexNet, the mean AUC and BAC values for 29 kinds of BSs used in this study were 0.710 ± 0.070 and 0.503 ± 0.210, respectively (Figure S5a,b). In addition, the maximum AUC and BAC values for the BSs indicated 0.857 at BS:5 and 0.796 at BSs:35 and 40, respectively (Figure S5a,b). It has been reported previously that the GoogLeNet used in this study showed higher AUC and BAC values than the AlexNet [49]. The mean AUC and BAC values for 29 kinds of BSs utilized in the GoogLeNet were 0.769 ± 0.038 and 0.737 ± 0.114, respectively (Figure S5a,b). The maximum AUC and BAC values in the GoogLeNet exhibited 0.886 at BS:1 and 0.819 at BS:1, respectively (Figure S5a,b). Therefore, the GoogLeNet was selected for the next analysis.

### 2.2. Contributions of Conformational Sampling of Chemical Compounds for Prediction Performance in the DeepSnap-DL Approach

**Figure 2:**
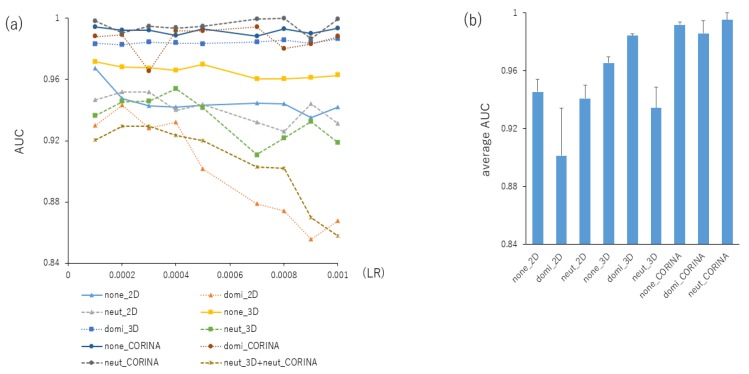
A contribution of the performance of prediction models with different wash conditions in the preparation of chemical structures of molecular operating environment (MOE) software. In the preparation for 3D chemical structures using MOE software, combinations of three kinds of protonation (none, dominate, and neutralize) and coordinates (2D, 3D, and CORINA) were utilized. The image produced by DeepSnap had the following angles and parameters: (176°, 176°, 176°), MPS:100, ZF:100, AT:23%, BR:14.5 mÅ, BMD:0.4 Å, BT:0.8 Å using nonoverlapped samples (Tra:Val:Test = 16:16:1) of the build DL-based prediction model by GoogLeNet from LR:0.001 to 0.0001 (**a**). The averages of AUCs of each LR were calculated (**b**).

Since numerous compounds appear in tautomertic forms in solution [50,51], it is difficult to describe a suitable molecular structure as a single variation of the structure. Thus, to investigate the contribution of conformational sampling of the 3D chemical structures to the prediction performance, the combinations of various cleaning rules, such as adjusting the protonation states (none, dominant, neutralize) and coordinating washed species (depict 2D, rebuild 3D, CORINA) in the MOE database wash treatment, were used to produce the 3D structures. Each AUC value for nine kinds of LRs from 0.0001 to 0.001 in ten kinds of cleaning rules, combining the protonation states and coordinating washed species, were calculated using the GoogLeNet (Figure 2a, Figure S6). The mean AUC values for the nine LRs were 0.945 ± 0.009 (none_2D), 0.901 ± 0.033 (domi_2D), 0.941 ± 0.009 (neut_2D), 0.965 ± 0.004 (none_3D), 0.984 ± 0.001 (domi_3D), 0.934 ± 0.014 (neut_3D), 0.992 ± 0.002 (none_CORINA), 0.986 ± 0.009 (domi_ CORINA), 0.995 ± 0.005 (neut_ CORINA), 0.906 ± 0.026 (neut_3D+neut_ CORINA) (Figure 2b). Next, to investigate the effect of dataset splits for the prediction performance using the neut_CORINA wash condition, a given dataset can be split into a total of 30 kinds of ratios for the Tra, Val, and Test datasets, in which Tra:Val:Test = X:X:1 or X:1:1, where X denote a variable integer (Table S5). The mean AUC and BAC values for 30 datasets were 0.999 ± 0.001 and 0.996 ± 0.003, respectively (Table S5). Additionally, the mean AUC and BAC values for Tra:Val:Test = X:X:1 or X:1:1 were AUC = 0.999 ± 0.001 (X:X:1) or 0.998 ± 0.002 (X:1:1) (Table S5) and BAC = 0.997 ± 0.003 (X:1:1) or 0.995 ± 0.004 (X:1:1) (Table S5). Meanwhile, to assess the contribution of the angles to the DeepSnap-DL for the prediction performance, a total of 16 angles from (360°, 360°, 360°) to (60°, 60°, 60°), corresponding to the number of pictures from one to 216, were analyzed for the production of pictures at three kinds of ratios with Tra:Val:Test = 8:8:1, 16:16:1, and 21:21:1 using the neut_CORINA wash condition (Table S6). The mean AUC and BAC values for 16 angles applied in the GoogLeNet were as follows: 0.9994 ± 0.0022 (8:8:1, AUC), 0.9996 ± 0.0012

(16:16:1,AUC), 0.9990 ± 0.0024 (21:21:1,AUC), 0.9965 ± 0.0092 (8:8:1,BAC), 0.9974 ± 0.0070 (16:16:1,BAC), and 0.9688 ± 0.1161 (21:21:1, BAC) (Table S6). Therefore, to investigate the contribution of conformational sampling to the prediction performance with the following angles (280°, 280°, 280°) in DeepSnap, the ten kinds of combinations of different cleaning rules were analyzed using two kinds of ratios with Tra:Val:Test = 1:1:1 and 4:4:1 (Table 1, Table S7). By considering the results with the following angles (176°, 176°, 176°), the neut_CORINA wash condition exhibited the highest performance: 0.998 ± 0.002 and 0.999 ± 0.001 (Tra:Val:Test = 1:1:1 and 4:4:1, AUC), 0.991 ± 0.004 and 0.993 ± 0.005 (Tra:Val:Test = 1:1:1 and 4:4:1, BAC), 0.991 ± 0.006 and 0.993 ± 0.005 (Tra:Val:Test = 1:1:1 and 4:4:1, Acc), 0.958 ± 0.024 and 0.969 ± 0.021 (Tra:Val:Test = 1:1:1 and 4:4:1, F), and 0.954 ± 0.026 and 0.966 ± 0.023 (Tra:Val:Test = 1:1:1 and 4:4:1, MCC) (Table 1, Table S7). Next, to study the contribution of the angles to the DeepSnap-DL for the prediction performance with split ratios of datasets, three kinds of angles (176°, 176°, 176°), (280°, 280°, 280°), and (360°, 360°, 360°) were used at eight kinds of ratios from Tra:Val:Test = 1:1:1 to 8:8:1 using the neut_CORINA wash condition (Table 2, Figure S7a–g). The prediction model quality and reliability depend on various conditions based on a similarity between the training and test datasets, including prediction space coverage, and applicability domain [52,53,54,55]. Thus, we split the datasets randomly shuffled into N groups, then took one group as the test dataset (hold out) to evaluate the model performance and the remaining groups as Tra or Val datasets. Moreover, for the CAR nonspecific activity scoring, scores datasets labeled randomly were utilized (Table 2, 280°PT in Figure S7). The performance at angles (176°, 176°, 176°) and (280°, 280°, 280°) significantly indicated high values, despite the differences of ratios with Tra:Val:Test (Figure S7 a–g). The performance at angles (360°, 360°, 360°) showed middle values among 280°PT, 176°, and 280° and increases along with ratio with Tra:Val (Table 2, Figure S7 a–g). The DL models have shown higher prediction performance and calculation costs than the traditional ML methods, such as RF and the SVM, due to the model structure complexity and gradient descent algorithm [56]. Furthermore, to analyze the combination of angles or the number of images in the DeepSnap with the prediction performance, a total of 14 kinds of combinations of pictures with various angles, including three kinds of picture numbers were utilized using the optimized parameters (Angle: 280°, MPS: 100, ZF: 100, AT: 23%, BR: 14.5 mÅ, BMD: 0.4 Å, BT: 0.8 Å, LR: 0.0008, BS: 108, GoogleNet) in the ratio with Tra:Val:Test = 1:1:1 (Table 2). The highest performance at four pictures, which include (0°, 0°, 0°), (280°, 0°, 0°), (0°, 280°, 0°), and (0°, 0°, 280°), was observed at 0.991 ± 0.003 (BAC), 0.971 ± 0.010 (F), and 0.967 ± 0.012 (MCC), respectively (Table 3, Table S8,). These findings indicate the possibility of reducing the calculation cost while maintaining high prediction performance.

Generally, the construction of quantitative structure–activity relationship (QSAR) prediction models with high performance could be disturbed when the endpoint is complex [57,58], or structurally similar molecules exhibit a large difference in potency, that is, activity cliffs, where small chemical modifications lead to profound effects on biological activity [59,60,61]. However, if there are a sufficient number of chemical compounds with structural quality that can show complex endpoints and slight chemical structural differences then QSAR analysis could overcame this issue. Since they represent the structural relationships between small chemical modifications and large potency, there may be principal indicators for QSAR if these chemical changes or substitution sites were identified during compound optimization. [38,62]. Following this perspective, the DeepSnap-DL approach that can extract an appropriate feature of a chemical structure using numerous input data for the DL may be considered as a QSAR approach, which shows high prediction performance. This is because the DL is enabled to automatically transform low-level features to higher and more abstract levels as a feature extractor and to learn numerous datasets [47,63]. In this study, there was a problem regarding the datasets from the Tox21 10k library used that imbalanced the activity scores of the CAR agonist (active: 10.8% and inactive: 89.2% in nonoverlapped sample, Table S3). To correctly evaluate the performance of prediction models constructed from the imbalanced datasets, we used the following as evaluation criteria: the BAC, F value, and MCC. To coordinate 3D structure preparation, high performances were observed at two different data split ratios with 1:1:1 or 4:4:1 when the CORINA on the DeepSnap-DL approach was employed, as demonstrated in Table 1. When these preparation procedures of the 3D chemical structure are applied, dataset imbalance and differences in chemica space coverage by different dataset splits seem not to adversely affect the robustness of the prediction model of CAR activity.

### 2.3. The Prediction Performance of the DeepSnap-DL Approach Compared with the Conventional ML

To evaluate the prediction performance of the DeepSnap appoach, two ML approaches, RF and extreme gradient boosting (XGB) were used to formulate the prediction models by applying the nonoverlapped chemical compounds library. By utilizing a molecular descriptor calculation software application, MORDRED, which is a non-copyleft open-source software, a total of 836 descriptors were extracted (Table S9a,b) [64]. This software has high flexibility, so that it can calculate uncommon-range descriptors without modifying the source code [64]. Thus, new molecular descriptors can be created by using the descriptor arithmetic feature. In addition, a total of 7140 chemical compounds for the CAR agonist split randomly into Tra and test datasets at a 1:1 ratio. Based on the quantitative descriptors for molecular structures extracted by MORDRED, the two classification and regression tools, RF and XGB, were applied to predict the compounds’ activity and build ten prediction models of RF and XGB, respectively, by optimizing parameters, including max_depth, nEstimators, and max_features (Table 4). The highest mean AUC values of RF and XGB in five independent tests were 0.8842 ± 0.0052 (max_depth:20, nEstimators:1000, and max_features:120) and 0.8890 ± 0.0072 (max_depth:3, nEstimators:5000, and max_features:60), respectively (Table 4). These results demonstrated that the DeepSnap-DL method outperformed the traditional ML methods for constructing the prediction model of the CAR agonist. However, the prediction performance by the RF and XGB showed a relatively high prediction ability. This result may suggest that adjustment of input data is a critical step in building a prediction model. Moreover, the feature extraction or their selection that was performed in the conventional ML methods can be automatically performed without human intervention in the DeepSnap-DL method, so the prediction model is expected to achieve high throughput and high performance. On the other hand, since a large amount of supervised data are required as learning data when building a prediction model, it might be necessary to reduce further the calculation cost.

## 3. Materials and Methods

### 3.1. Data

In an approach reported previously [45], a total of 9523 chemical structures and the corresponding CAR activity scores were downloaded in the simplified molecular input line entry system (SMILES) format from the PubChem database (AID 1224892), derived from the Tox21 10k library (Table S3). The library includes some chemical compounds that are the same but with different activity scores, which represent an under or over 40 score, as different ID numbers. Therefore, these chemical compounds with indefinite activity criteria and/or with nonorganic compounds were eliminated, and a total of 7141 chemicals for the CAR were selected as nonoverlapped input data (Table S3). In the primary screening of the Tox21 program, the CAR activity scores were represented from 0% to 100% based on a compound concentration response analysis as follows: % Activity = ((Vcompound–Vdmso)/(Vpos–Vdmso)) × 100, where Vcompound, Vdmso, and Vpos denote the compound, the median values of the DMSO only, and the median value of the positive control well values in the reporter gene assay, respectively, which were then corrected by utilizing compound-free control plates, that is, DMSO-only plates, at the beginning and end of the compound plate measurement [18,65]. Concentration response titration points for each compound were fitted to a four-parameter Hill equation, which yields the concentrations of half-maximal activity (AC50) and maximal response (efficacy) values [18,66]. The activity scores were grouped into the following three classes: (1) zero, (2) from 1 to 39, and (3) from 40 to 100, represented as inactive, inconclusive, and active compounds, respectively. This study, defines active or inactive compounds from 40 to 100 or from 0 to 39 of the activity score (Table S3). For the permutation test for the nonspecific activity scoring of CAR, activity scores labeled randomly to chemical compounds were applied. Then, a MOE 2018 application software program (MOLSIS Inc., Tokyo, Japan) was utilized to produce the molecular geometry replaced by lower-energy 3D coordinates with rotatable torsions per compound, which was optimized by a two-step method: a cyclic embedder based on distance geometry and refinement (called rebuild 3D), in which the washed species are to be replaced by those generated by a cyclic 3D embedder. Thus, using the external program, CORINA classic software (Molecular Networks GmbH, Nürnberg, Germany, https://www.mn-am.com/products/corina) defines a single sTable conformation, and the 3D structures are saved finally in the SDF file format, as described previously [45,67,68]. Besides, if depict 2D is chosen, the coordinates of the washed species will be replaced by the results of the 2D depiction layout algorithm. In addition, to investigate whether each structure is in a suitable form for subsequent KEs, which are triggered by MIEs, the protonation states were adjusted. Charged species will be replaced with the following if the protonation menu was set to neutralize: (1) all the atoms are neutral; (2) the species is neutral overall; and (3) the least charge-bearing form of the structure. If it was set to dominant, the molecule was replaced with the dominant promoter/tautomer at a specified pH of pH: 7 used in this study. The ten types of combinations of the protonation states as follows: (none, dominant, neutralize) and coordinating washed species (depict 2D, rebuild 3D, CORINA) in washing the MOE database were investigated; none_2D (none, depict 2D), domi_2D (dominant, depict 2D), neut_2D (neutralize, depict 2D), none_3D (none, rebuild 3D), domi_3D (dominant, rebuild 3D), neut_3D (neutralize, rebuild 3D), none_CORINA(none, CORINA), domi_ CORINA (dominant, CORINA), neut_ CORINA (neutralize, CORINA), neut_3D + neut_CORINA (meutralize, rebuild 3D, and then neutralize, CORINA).

### 3.2. DeepSnap

The 3D chemical structures were depicted as 3D ball-and-stick models with different colors corresponding to different atoms by a Jmol, which is an open-source Java viewer for 3D structures [69,70,71]. They were captured automatically as snapshots in selected angle increments on the *x*-, *y*-, and *z*-axes, which were saved as 256 × 256 pixel resolution PNG files (RGB) and split into three types of datasets, that is training (Tra), validation (Val), and test (Test) datasets, as depicted in Figure S8 [44,45]. During the DeepSnap depiction process, some parameters were set to design suitable molecular images for their classifications at the next step, such as image pixel size, image format (png or jpg), molecule number per SDF file to split into MPS, zoom factor (ZF, %), atom size for van der Waals radius (AT, %), bond radius (BR, mÅ), minimum bond distance (MBD), and bond tolerance (BT).

### 3.3. ML Models

The following three different ML models were chosen to construct the prediction models for CAR activity: (1) DL, (2) RF, and (3) extreme gradient boosting (XGBoost, which we denote as XGB). For the DL, all the 2D-PNG image files produced by DeepSnap were resized by utilizing NVIDIA DL GPU Training System (DIGITS) version 4.0.0 software (NVIDIA, Santa Clara, CA, USA), which was constituted based on managing data, designing, and training NNs on four-GPU systems, Tesla-V100-PCIE (31.7GB); real-time monitoring for model performance; and selecting the best performance model from the results [49] with a resolution of 256 × 256 pixels as input data. To rapidly train and fine-tune the highly accurate deep neural networks (DNNs) using the input Tra and Val datasets based on the image classification and building the prediction model as transfer learning, a pretrained open-source DL model, Caffe, and the open-source software on the CentOS Linux distribution 7.3.1611 were used. AlexNet is a CNN developed by the University of Toronto, as described in a previous study [72]. The CNN architecture comprises eight pretrained layers, which include five convolutional/pooling layers that convert the feature volume and reduce the layers. This was achieved by compressing images using max-pooling compresses and selecting the maximum value in each region as a representative value. Convolutional/pooling layer I converted the previous volume (224 × 224 × 3) to (11 × 11 × 3); convolutional/pooling layers II, III, IV, and V converted the results of layers I, II, III, and IV to (5 × 5 × 48), (3 × 3 × 256), (3 × 3 × 192), and (3 × 3 × 192), respectively. These fully connected layers make final connections between feature values and forces that were converted to zero to suppress overfitting (dropout) by a total of 4,096 neurons (Figure S9) [72,73,74]. This study utilized a GoogLeNet model that comprised 22 complex and deep CNN architectures called “Inception”, which concatenates different filter sizes and dimensions into a single new filter and introduces sparsity and multiscale information in one block; and it comprises two convolutional layers, two pooling layers, and nine “Inception” modules, in which each module has six convolution layers and one pooling layer (Figure S10) [49]. The filters and weights of the GoogLeNet were iteratively discriminated via error back-propagation that can convert a loss into gradients to rectify the last layer and calculate the correcting gradients [73,75]. For the RF and XGB, molecular descriptors were calculated using a Python package called Mordred (https://github.com/mordred-descriptor/mordred) [64]. Classification experiments were conducted in the Python programming language using specific classifier implementations, RF, and XGB provided by the scikit-learn and rdkit Python packages [76,77,78,79].

### 3.4. Evaluation of the Predictive Model

Using the external test dataset in the DL prediction model, the probability of the prediction results was analyzed by applying the designed prediction models. Since we calculated the probabilities for each image prepared from different angles with the *x*-, *y*-, and *z*-axes directions calculated for one molecule during the process of the DeepSnap-DL method, the medians of these predicted values were used as the representative values for target molecules, as described previously [80]. Based on the sensitivity, which is a true positive rate identified as positive for all the positive samples including true and false positives, and the specificity, which is a true negative rate identified as negative for all the negative samples including true and false negatives, a confusion matrix regarding the predicted and the experimentally defined labels was used to make the ROC curve and calculate the AUC using JMP Pro 14, which is a statistical discovery software (SAS Institute Inc., Cary, NC, USA), as reported previously [45]. Thus, it follows that where TP, FN, TN, and FP denote true positive, false negative, true negative, and false positive, respectively:

Sensitivity = ΣTPs/(ΣTPs + ΣFNs),(1)

Specificity = ΣTNs/(ΣTNs + ΣFPs).(2)

Additionally, since the proportion between the “active” and “inactive” compounds for the activity scores is biased in the library [18], the balanced accuracy (BAC), accuracy in test datasets (Acc), F value, and Matthews correlation coefficient (MCC) were utilized to avoid overfitting by applying a cut-off point calculated using the JMP Pro 14 and statistical discovery software. Thus, it follows that

BAC = (sensitivity + specificity)/2(3)

Accuracy = ((TP + TN)/(TP + FP + TN + FN)(4)

Precision = TP/(TP + FP)(5)

Recall = TP/(TP + FN)(6)

F value = 2 × Recall × Precision/(Recall + Precision)(7)

(8) $$\mathrm{MCC}\;=\;(\mathrm{TP}\times \mathrm{TN}\;-\;\mathrm{FP}\times \mathrm{FN})/\sqrt{\left\{\left(\mathrm{TP}+\mathrm{FP}\right)\times \left(\mathrm{TP}+\mathrm{FN}\right)\times \left(\mathrm{TN}+\mathrm{FP}\right)\times \left(\mathrm{TN}+\mathrm{FN}\right)\right\}}.$$

For RF and XGB, AUC was calculated by utilizing the scikit-learn Python packages [77]. Prediction models were optimized by possible important parameters (https://scikit-learn.org/stable/modules/generated/sklearn.ensemble.RandomForestClassifier.html) [80,81,82].

## 4. Conclusions

This study proposed a strategy based on a DL model that can predict the CAR agonist, which has been studied extensively by applying the DeepSnap-DL approach using the Tox21 10k library, due to its importance in toxicological and pharmaceutical fields. When compared with the other conventional ML methods, which include RF and Xgboost, the results indicated that the DeepSnap-DL approach is superior in prediction performance using the suitable 3D chemical structures produced by CORINA as input data for the DL. Based on the DeepSnap-DL approach, a similar strategy could also be applied to other MIE targets and KE pathways to understand the mechanisms in which chemical toxicants cause AO. Generally, we conclude that the optimal utilization of 3D structures in the DeepSnap-DL approach could demonstrate a prediction model with a very high performance in regards to the CAR agonist.

## Figures and Tables

**Table 1:** Prediction performances with different preparations of chemical structures in the DeepSnap.

					AUC		Acc		MCC	
Train:val:test	Protonation	Coordinate	Protonation	Coordinate	Average	SD	Average	SD	Average	SD
1:1:1	none	2D			0.930	0.006	0.967	0.007	0.821	0.035
1:1:1	dominate	2D			0.904	0.011	0.926	0.048	0.668	0.131
1:1:1	neutralize	2D			0.890	0.006	0.919	0.032	0.619	0.115
1:1:1	none	3D			0.907	0.008	0.797	0.035	0.440	0.019
1:1:1	dominate	3D			0.971	0.003	0.927	0.001	0.734	0.005
1:1:1	neutralize	3D			0.924	0.007	0.969	0.003	0.827	0.017
1:1:1	none	CORINA			0.989	0.003	0.958	0.003	0.826	0.012
1:1:1	dominate	CORINA			0.996	0.002	0.982	0.005	0.914	0.021
1:1:1	neutralize	CORINA			**0.998**	0.002	0.991	0.006	0.954	0.026
1:1:1	neutralize	3D	neutralize	CORINA	0.798	0.016	0.707	0.020	0.302	0.018
4:4:1	none	2D			0.923	0.024	0.959	0.029	0.798	0.107
4:4:1	dominate	2D			0.906	0.013	0.894	0.069	0.609	0.139
4:4:1	neutralize	2D			0.898	0.019	0.903	0.059	0.621	0.125
4:4:1	none	3D			0.911	0.009	0.801	0.043	0.458	0.033
4:4:1	dominate	3D			0.972	0.003	0.928	0.012	0.739	0.030
4:4:1	neutralize	3D			0.927	0.011	0.971	0.002	0.839	0.010
4:4:1	none	CORINA			0.990	0.003	0.957	0.009	0.821	0.029
4:4:1	dominate	CORINA			0.997	0.001	0.985	0.003	0.927	0.015
4:4:1	neutralize	CORINA			**0.999**	0.001	**0.993**	0.005	**0.966**	0.023
4:4:1	neutralize	3D	neutralize	CORINA	0.802	0.014	0.684	0.043	0.311	0.021

Parameters (angle: 280, MPS: 100, ZF:100, AT: 23%, BR: 14.5 mÅ, BMD: 0.4 Å, BT: 0.8 Å, LR: 0.0008, BS: 108, GoogleNet), *n* = 3 or 9 for 1:1:1 or 4:4:1, respectively. Maximum values for AUC, Accuray in test dataset (Acc), and Matthews correlation coefficient (MCC) in each dataset are indicated by bold.

**Table 2:** Prediction performances with different angles in the DeepSnap.

			176°		280°		360°		280°PT	
	Train:Val:Test	N	Average	SD	Average	SD	Average	SD	Average	SD
AUC	1:1:1	3	1.000	0.000	0.998	0.002	0.932	0.027	0.537	0.009
	2:2:1	5	0.999	0.001	0.998	0.001	0.964	0.005	0.522	0.013
	3:3:1	6	0.999	0.000	0.998	0.001	0.972	0.009	0.544	0.019
	4:4:1	9	0.998	0.003	0.999	0.001	0.979	0.005	0.545	0.027
	5:5:1	11	0.998	0.003	0.998	0.002	0.983	0.005	0.534	0.016
	6:6:1	13	0.999	0.001	0.998	0.002	0.983	0.008	0.529	0.022
	7:7:1	15	0.998	0.002	0.998	0.002	0.982	0.007	0.555	0.043
	8:8:1	17	0.999	0.003	0.998	0.003	0.983	0.009	0.552	0.044
Acc	1:1:1	3	0.997	0.001	0.991	0.006	0.851	0.037	0.422	0.009
	2:2:1	5	0.995	0.002	0.993	0.005	0.898	0.005	0.554	0.013
	3:3:1	6	0.993	0.006	0.988	0.008	0.918	0.034	0.555	0.019
	4:4:1	9	0.995	0.003	0.993	0.005	0.925	0.020	0.449	0.027
	5:5:1	11	0.993	0.004	0.992	0.004	0.934	0.022	0.507	0.016
	6:6:1	13	0.995	0.002	0.993	0.007	0.942	0.022	0.498	0.022
	7:7:1	15	0.994	0.003	0.993	0.007	0.934	0.030	0.513	0.043
	8:8:1	17	0.996	0.003	0.992	0.009	0.931	0.049	0.527	0.044
MCC	1:1:1	3	0.986	0.006	0.954	0.026	0.547	0.074	0.018	0.073
	2:2:1	5	0.977	0.012	0.966	0.022	0.647	0.016	0.018	0.047
	3:3:1	6	0.966	0.028	0.942	0.037	0.705	0.074	0.025	0.065
	4:4:1	9	0.976	0.015	0.966	0.023	0.723	0.049	0.078	0.022
	5:5:1	11	0.967	0.017	0.962	0.018	0.749	0.055	0.057	0.055
	6:6:1	13	0.976	0.012	0.970	0.028	0.768	0.060	0.062	0.049
	7:7:1	15	0.970	0.012	0.966	0.031	0.755	0.072	0.069	0.079
	8:8:1	17	0.978	0.016	0.961	0.041	0.749	0.103	0.060	0.092

Parameters (MPS: 100, ZF: 100, AT: 23%, BR: 14.5 mÅ, BMD: 0.4 Å, BT: 0.8 Å, LR: 0.0008, BS: 108, GoogleNet). Protonation: neutralize, coordinate: CORINA, train:val:test: ratio of train, validation, and test datasets, n: number of external test datasets, average: means of Accuray in test dataset (Acc), AUC, and Matthews correlation coefficient (MCC) for n, sd: standard deviations of MCC for n, 280°PT: permutation test for activity scores at 280° angle.

**Table 3:** Prediction performances with combinations of different angles in the DeepSnap.

	Angles on *x*-, *y*-, *z*-axes				AUC		Acc		MCC	
No. of Picture	Pic1	Pic2	Pic3	Pic4	Average	SD	Average	SD	Average	SD
4	0,0,0,	280,0,0,	0,280,0	0,0,280	0.999	0.000	0.994	0.002	0.967	0.012
4	280,280,280,	0,280,280,	280,0,280	280,280,0	0.998	0.002	0.988	0.004	0.941	0.021
4	0,0,0,	0,280,280,	280.0.280	280,280,0	0.998	0.001	0.990	0.003	0.952	0.014
4	0,0,0,	280,0,0,	280.0.280	280,280,0	0.997	0.003	0.988	0.006	0.943	0.027
4	0,0,0,	280,0,0,	0.280.0	280,280,0	0.996	0.002	0.991	0.004	0.953	0.018
3	-	280,0,0,	0,280,0	0,0,280	0.995	0.004	0.984	0.006	0.921	0.027
3	0,0,0,	-	0,280,0	0,0,280	0.998	0.001	0.987	0.005	0.935	0.024
3	0,0,0,	280,0,0,	-	0,0,280	0.998	0.001	0.988	0.008	0.943	0.037
3	0,0,0,	280,0,0,	0,280,0	-	0.995	0.002	0.984	0.007	0.921	0.032
2	0,0,0,	280,0,0,	-	-	0.995	0.002	0.976	0.012	0.890	0.048
2	0,0,0,	-	0,280,0	-	0.993	0.002	0.970	0.015	0.864	0.055
2	0,0,0,	-	-	0,0,280	0.996	0.000	0.978	0.009	0.896	0.034
2	-	280,0,0,	0,280,0	-	0.982	0.008	0.960	0.006	0.817	0.010
2	-	-	0,280,0	0,0,280	0.998	0.001	0.986	0.002	0.931	0.010

Parameters (angle: 280, MPS: 100, ZF: 100, AT: 23%, BR: 14.5 mÅ, BMD: 0.4 Å, BT: 0.8 Å, LR: 0.0008, BS: 108, GoogleNet). Parameters (angle: 280, MPS: 100, ZF: 100, AT: 23%, BR: 14.5 mÅ, BM: 0.4 Å, BT: 0.8 Å, LR: 0.0008, BS: 108, GoogleNet). Maximum values for AUC, Accuracy in test dataset (Acc), and Matthews correlation coefficient (MCC) are indicated by bold. Wash in MOE (protonation states: neutralize, coordinating washed species: CORINA).

**Table 4:** Prediction performances in extreme gradient boosting (XGB) and random forest (RF).

	Auc			Parameters	
Model #	Average	SD	Max_Depth	Nestimators	Max_Features
XGB_1	0.8855	0.0071	3	100	29
XGB_2	0.8862	0.0095	3	500	29
XGB_3	0.8854	0.0073	3	1000	29
XGB_4	0.8885	0.0033	3	5000	29
XGB_5	0.8883	0.0040	30	1000	29
XGB_6	0.8872	0.0089	3	5000	40
XGB_7	0.8851	0.0026	3	5000	50
XGB_8	**0.8890**	0.0072	3	5000	60
XGB_9	0.8873	0.0069	3	5000	100
XGB_10	0.8835	0.0075	3	5000	120
RF_1	0.8069	0.0193	2	10	29
RF_2	0.8314	0.0287	2	100	29
RF_3	0.8416	0.0252	2	1000	29
RF_4	0.8803	0.0053	20	1000	29
RF_5	0.8781	0.0104	200	1000	29
RF_6	0.8780	0.0083	20	5000	29
RF_7	0.8702	0.0067	20	1000	5
RF_8	0.8813	0.0032	20	1000	80
RF_9	0.8842	0.0052	20	1000	120
RF_10	0.8807	0.0055	20	1000	250

Average: means of AUCs for 5 tests. SD: standard deviations of AUCs for 5 independent tests. Maximum values for AUC in each models are indicated by bold.

## Supplementary Materials

Supplementary materials can be found at https://www.mdpi.com/1422-0067/20/19/4855/s1.

## Abbreviations

2D	Two-dimensional
3D	Three-dimensional
Acc	Accuracy in the test dataset
AO	Adverse outcome
AOP	Adverse outcome pathway
AUC	Area under the curve
AT	Atom size for van der Waals
AV	Accuracy in the validation dataset
BAC	Balanced accuracy
BMD	Bound minimum distance
BR	Bond radius
BSs	Batch sizes
BT	Bond tolerance
CAR	Constitutive androstane receptor
CNN	Convolutional neural network
DIGITS	Deep learning GPU training system
DL	Deep learning
DNNs	Deep neural networks
F	F value
KEs	Key events
KER	Key event relationship
LR	Learning rate
LV	Loss in the validation dataset
MBD	Minimum bond distance
MCC	Matthews correlation coefficient
MIE	Molecular initiating event
ML	Machine learning
MOE	Molecular operating environment
MPS	Number of molecules per SDF file to split into
NN	Neural network
RF	Random Forest
ROC	Receiver operating characteristic
SMILES	Simplified molecular input line entry system
SVM	Support vector machine
Tox21	Toxicology in the 21st century
XGB	eXtreme Gradient Boosting
ZF	Zoom factor
