# Supplementary material for: Prediction Model with High-Performance Constitutive Androstane Receptor (CAR) Using DeepSnap-Deep Learning Approach from the Tox21 10K Compound Library
Source: Int J Mol Sci. 2019 Sep 30;20(19):4855. doi: 10.3390/ijms20194855 (PMC6801383; doi:10.3390/ijms20194855)
Supplement: Supplementary file 1 [file ijms-20-04855-s001.pdf]

Prediction Model with High-Performance Constitutive Androstane Receptor (CAR) Using DeepSnap-Deep Learning Approach from the Tox21 10K Compound Library

Yasunari Matsuzaka, Yoshihiro Uesawa

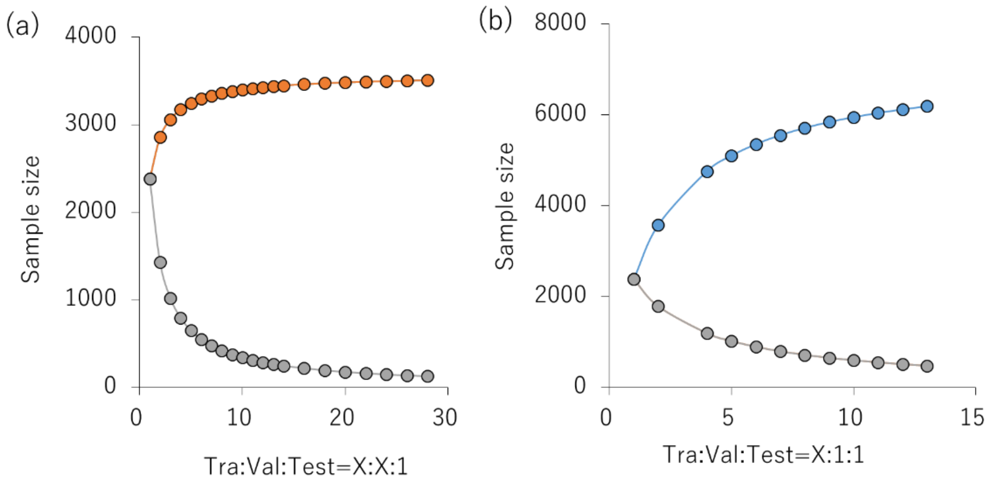

Figure S1. Sample size of train (Tra), validation (Val), test (Test) datasets. Total 7,141 of chemical compounds were split into three type of datasets, Tra, Val, and Test with twenty-one kinds of ratio from 1:1:1 to 28:28:1 (**a**) and eleven kinds of ratio from 2:1:1 to 13:1:1 (**b**).

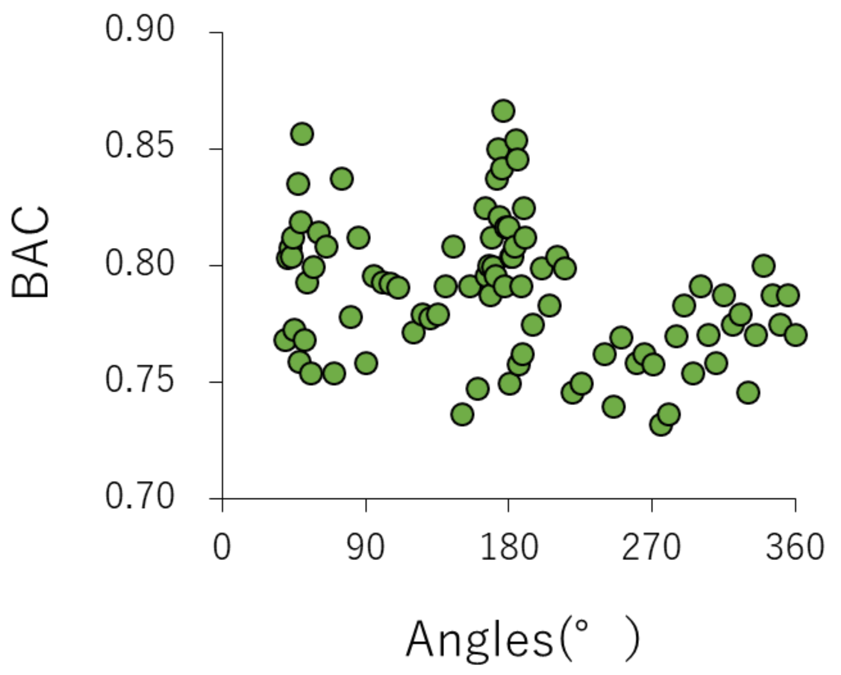

Figure S2. A contribution of performance of prediction models with angles of production of pictures in the DeepSnap approach: BAC, which are calculated by the DL- build prediction models in GoogLeNet using training, validation, and external test datasets produced by the DeepSnap approach with 92 and 53 different angles from (360° , 360° , 360°) to (38° , 38° , 38°) and from (360° , 360 ° , 360°) to (90° , 90° , 90°), with MPS:100, ZF:100, AT:23%, BR:21.1 mÅ, BMD:0.4 Å, BT:0.8 Å, LR:0.01, and BS:default.

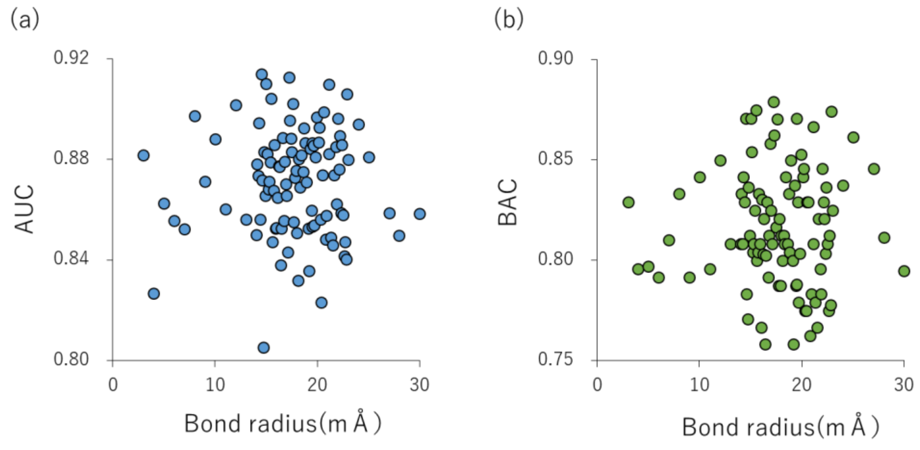

Figure S3. Contribution of performance of prediction model with BRs for production of image pictures in DeepSnap. (**a**) AUC and (**b**) BAC calculated by the DL-build prediction models in GoogLeNet using non-overlapped datasets for one hundred one of kinds of different BRs from 3mÅ to 30mÅwith angle:(176° , 176° , 176°), MPS:100, ZF:100, AT:23%, BMD:0.4Å, BT:0.8Å, LR:0.01, BS:default.

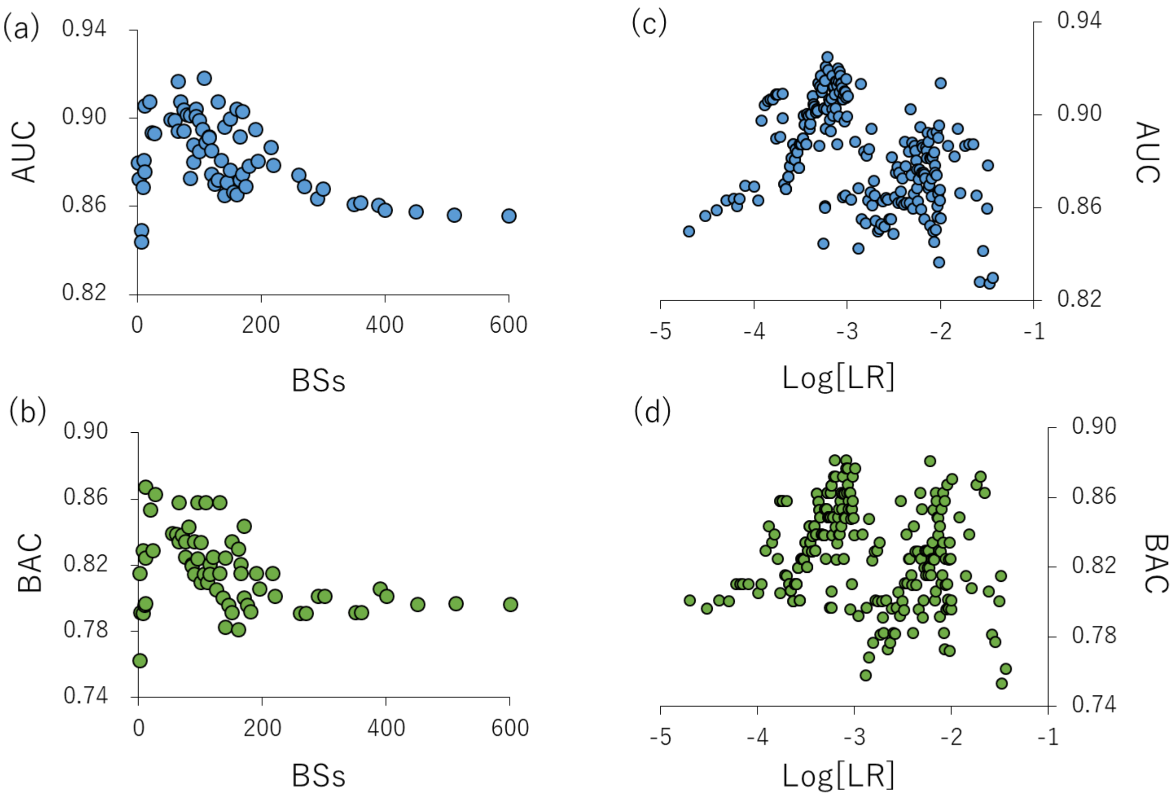

Figure S4. Contribution of performance of prediction models with BSs (**a**, **b**) and LRs (**c**,**d**). (**a**,**c**) AUC and (**b**,**d**) BAC calculated by the DL-build prediction models in GoogLeNet with LR: 0.0011 (a,b) and BS: default (c,d) using image pictures produced from non-overlapped samples by DeepSnap with angle: (176°, 176°, 176°), MPS:100, ZF:100, AT:23%, BR:14.5mÅ, BMD:0.4Å, BT:0.8Å.

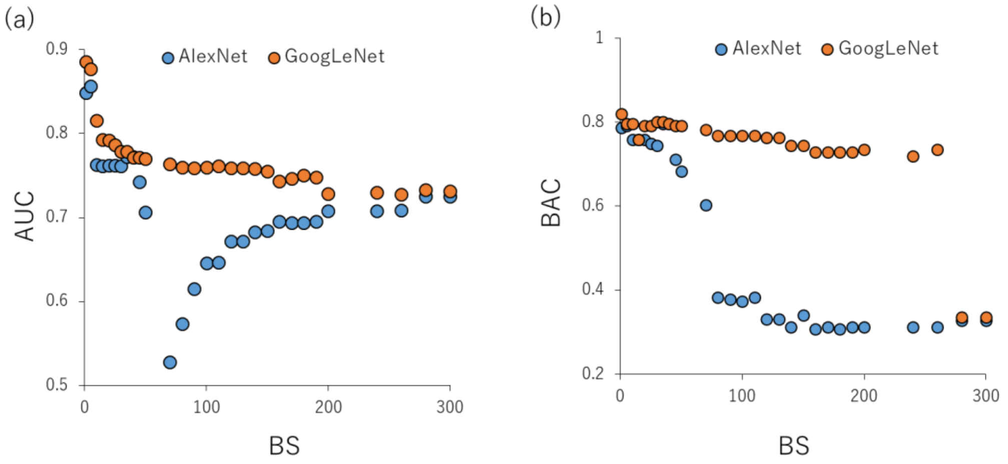

Figure S5. Comparison of performance of prediction models in AlexNet and GoogLeNet. Image pictures were produced by DeepSnap with angle: (176°, 176°, 176°), MPS:100, ZF:100, AT:23%, BR:14.5mÅ, BMD:0.4Å, BT:0.8Å, LR:0.00061 using non-overlapped samples. AUC (**a**) and BAC (**b**) were calculated by the DL-build prediction models using twenty-nine kinds of BSs from 1 to 300 with LR:0.00061 in AlexNet and GoogLeNet.

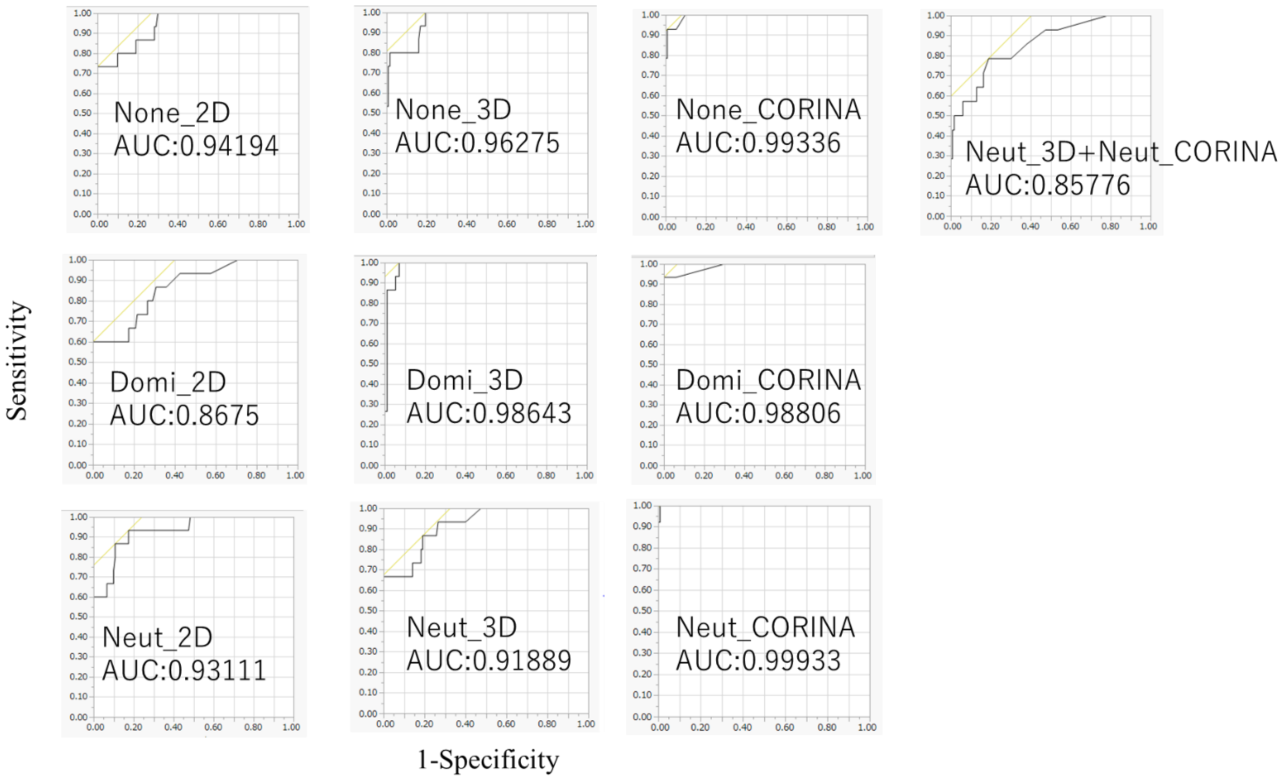

Figure S6. ROC of prediction models in different wash conditions in preparation of chemical structures of MOE software. Image pictures were produced by DeepSnap with angle: (176°, 176°, 176°), MPS:100, ZF:100, AT:23%, BR:14.5mÅ, BMD:0.4Å, BT:0.8Å, LR:0.001 using non-overlapped samples (Tra:Val:Test=16:16:1) and GoogLeNet. AUCs were indicated under each wash conditions.

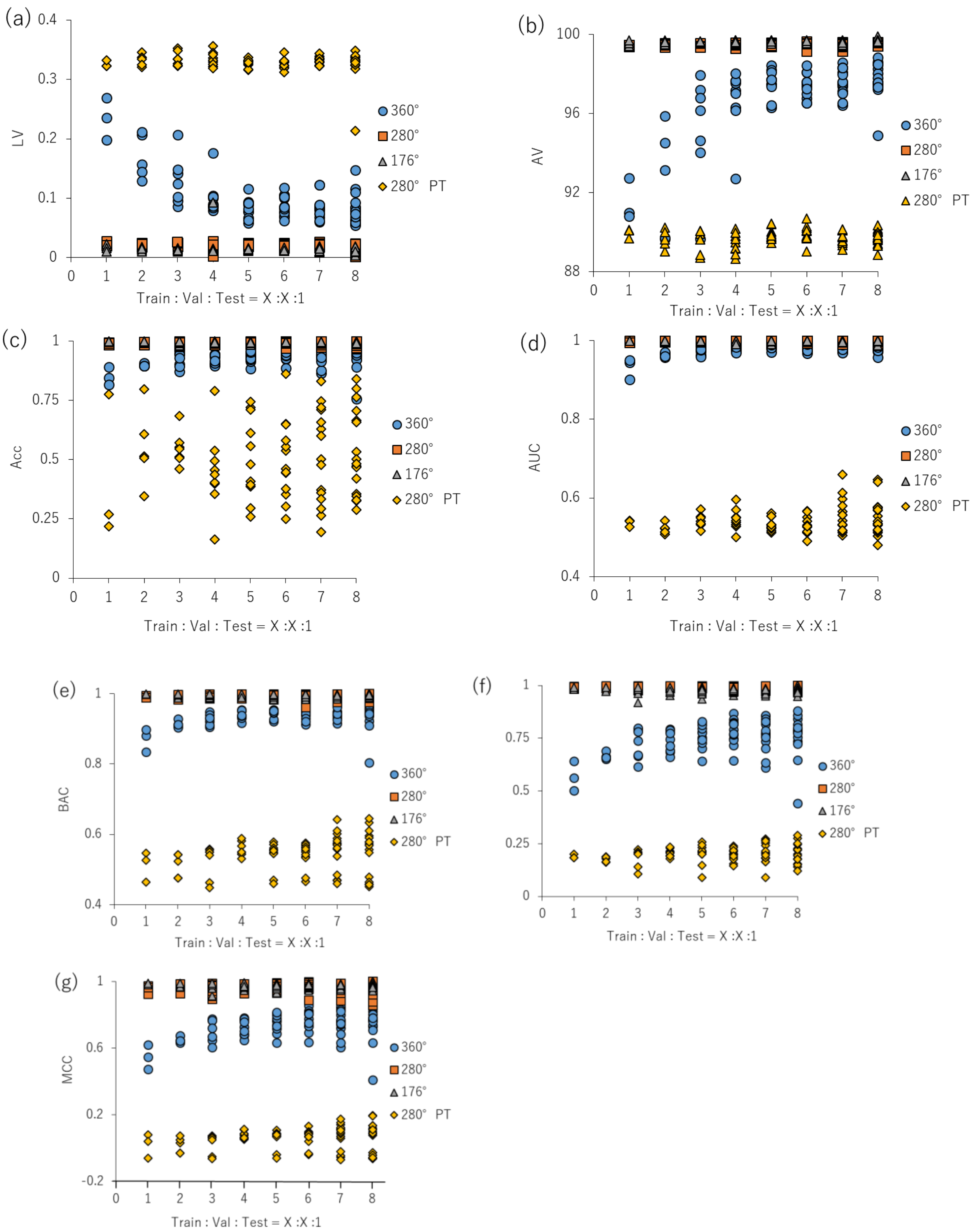

Figure S7. A contribution of the performance of prediction models with dataset split ratio and angles. (**a**) LV, (**b**) AV, (**c**) Acc, (**d**) AUC, (**e**) BAC, (**f**) F, and (**g**) MCC were calculated by applying the DL-build prediction models in GoogLeNet with eight kinds of ratios of Tra:Val:Test from 1:1:1 to 8:8:1, where X denotes a variable integer using pictures produced from the non-overlapped samples by DeepSnap with three kinds of angles and the following parameters: (176° , 176° , 176°), (280° , 280° , 280°), (360° , 360° , 360°), MPS:100, ZF:100, AT:23%, BR:14.5 mÅ, BMD:0.4 Å, BT:0.8 Å, LR:0.0008, and BS:108. 280° PT shows a permutation test with randomly labeled activity scores that are non-specific for CAR activity. N is equal to numbers of external test, i.e., 3, 5, 7, 9, 11, 13, 15, and 17 for eight kinds of ratios of Tra:Val:Test from 1:1:1 to 8:8:1, respectively.

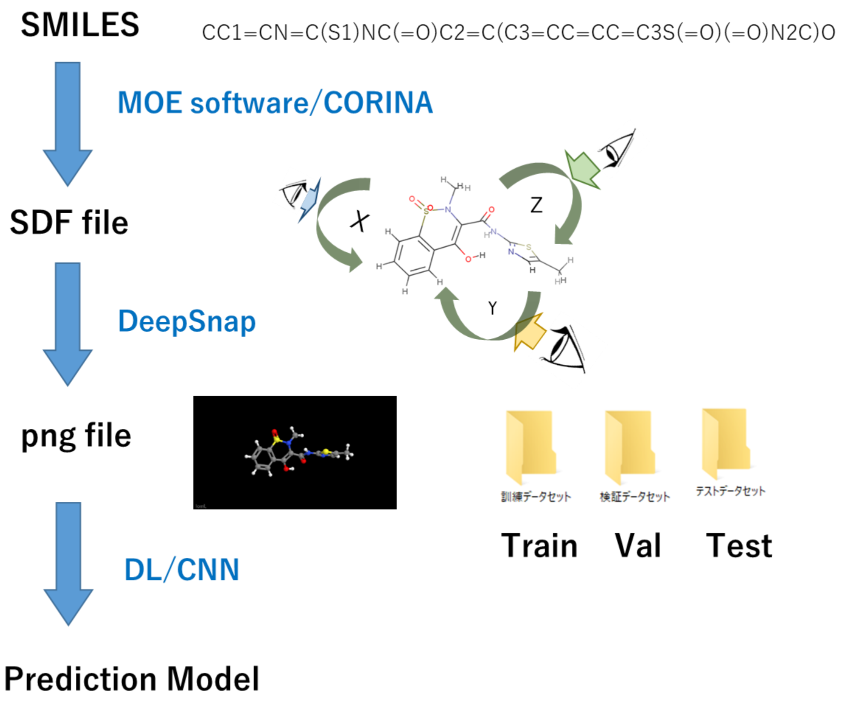

Figure S8. A schematic diagram of the DeepSnap-DL procedure. The chemical structure in the SMILES format is imported by CORINA classic software with washing in the MOE application into a 3D- structure in the SDF file format, which is photographed at an arbitrary angle on the x-, y-, and z-axes by applying Jmol- DeepSnap, and then the 2D- chemical image data produced are saved as PNG files in three datasets (training, validation, and test). From the image data utilized as the input dataset for the DL, the feature values were extracted automatically using a CNN, and the prediction model was finally built.

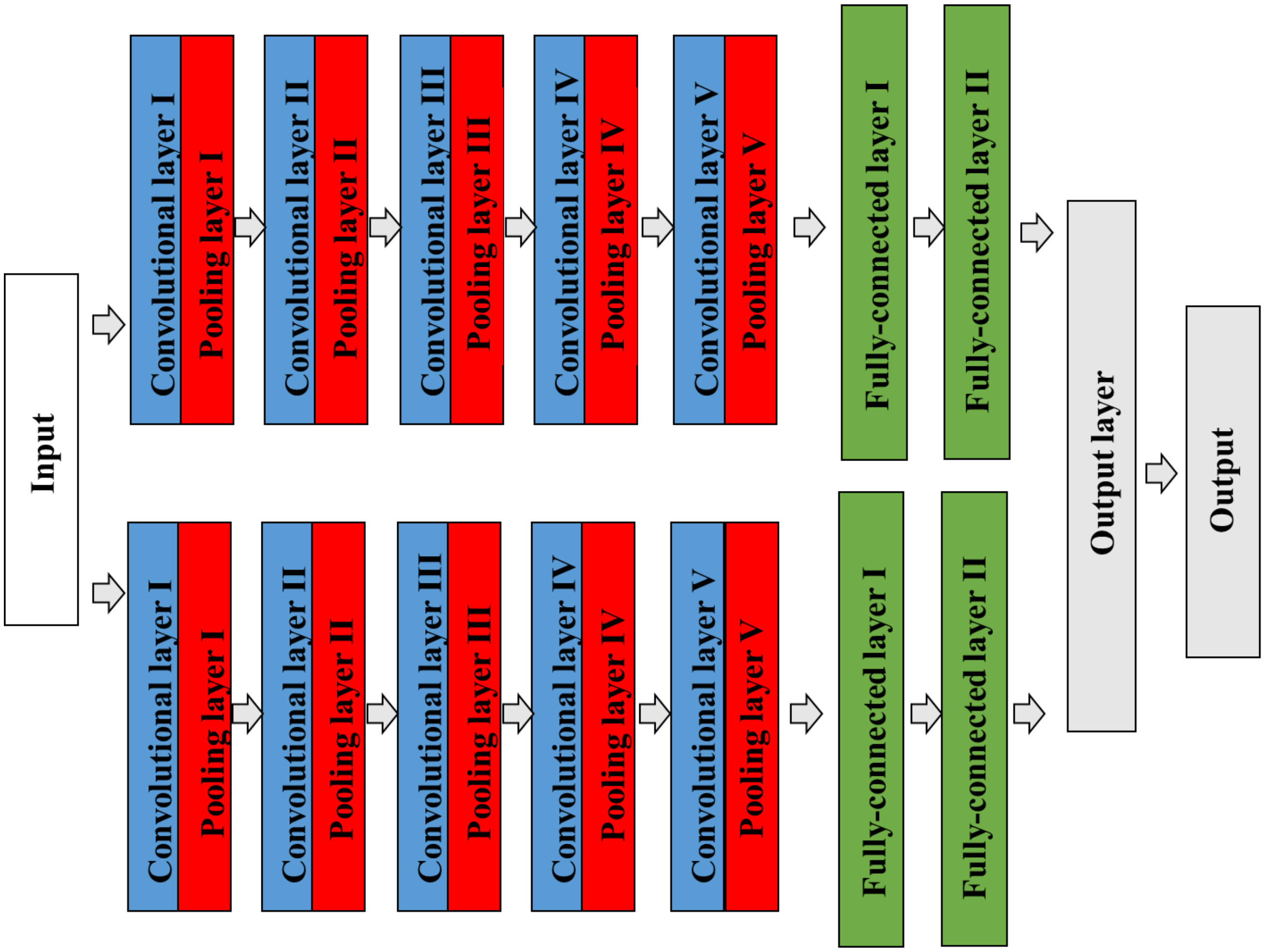

Figure S9. The architecture of the CNN model in AlexNet. The CNN contains a total of eight pre-learned layers, which consisted of five convolutional and max- pooling layers, three fully- connected layers, dropout, data augmentation, rectified linear unit activations, and stochastic gradient descent with momentum, including a total of 60 million parameters. The two adjacent convolutional and pooling layers are finally combined into the third fully connected layer.

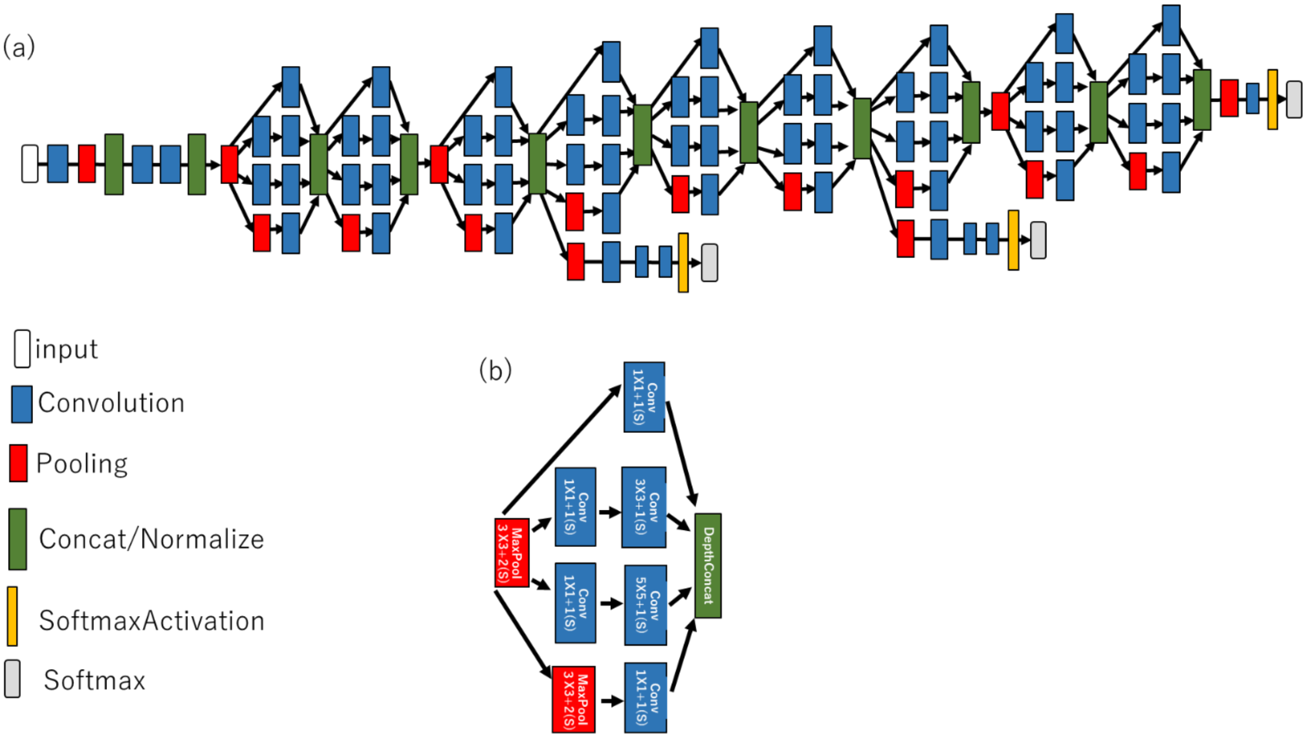

Figure S10. The architecture of the CNN model in GoogLeNet. The pre-trained CNN comprises a 22-layer DNN: (**a**) implemented with a novel element that is dubbed an inception module; and (**b**) implemented with batch normalization, image distortions, and RMSprop, including a total of 4 million parameters.
